# Notes and Comments

**Published:** 1898-12

**Authors:** 


### Notes and Comments.[1]

Production and Use of Carborundum.—The *Scientific American* says the Carborundum Company reports to us that its works have produced during the year 1896, in round numbers, 1,191,000 pounds, or 595½ tons, of crystalline carborundum. Says the *Engineering Mining Journal:* Consideration at the present is given to the production in crystalline form only, but another important industry in which carbide of silicon promises usefulness of the material. Some mention has been made of the experiments showing that carborundum can be used, and will, in all probability, take the place of ferro-silicon in the manufacture of steel. Professor Luehr-

[1] The assistant editor solicits contributions for this department,—new methods, new remedies and formulas, or any short practical note which may prove of value to the practitioner or student. Address 1338 Walnut Street, Philadelphia.

mann, of Germany, recently wrote an article on this subject, indicating that in the use of carborundum there will be in Germany alone, approximately, two thousand five hundred tons consumed annually, provided its cost would not exceed six cents a pound. It may be used for this purpose in an amorphous form, and the Carborundum Company is prepared to furnish it at a price slightly under this figure.

---

Tooth Extraction for Blindness.—A medical journal reports a case of blindness caused by crowded teeth. The patient, a boy of eleven years, was suddenly stricken blind. Several physicians were called in consultation, and one of them, happening to notice the crowded condition of the lad's teeth, advised the extraction of several of the teeth, stating that upon such action depended the child's restoration to sight. This advice was acted upon, and in a few days the boy's sight was completely restored.—*Dental Headlight.*

---

Liquefied Air as a Beverage.—The Paris correspondent of the *British Medical Journal* states that at a public dinner in Paris the other day, at which M. d'Arsonval was present, the guests were astonished by having liquefied air poured into their glasses of champagne. A year ago the Emperor of Germany was offered a glass of liquefied air. He raised the glass in honor of science, but refrained from putting it to his lips; the liquefied air in it would have burned them like hot coals. The liquefied air poured into the champagne became dispersed in white clouds, and mingled with the surrounding atmosphere. A bottle of air, if liquefied, can bear a transit of sixty hours without volatilization taking place.—*Philadelphia Medical Journal.*

---

Iodine and Permanganate of Potassium Stains.—Answering the inquiry for colorless iodine, I would give the following: Add a saturated solution of hyposulphite of soda to tincture of iodine slowly, and stir till the color disappears. No doubt in decolorizing in any manner there is a new chemical composition, but whether this affects the action of the original tincture or not is a question. To remove stains of tincture of iodine from clothing, skin, etc., the hyposulphite, of course, acts beautifully.

For removing the stains of permanganate of potassium from the hands or clothing, peroxide of hydrogen acts like magic.—Bret Black., M.D., in *Medical World.*

Quick Flasking.—Dr. B. H. Teague, in the *Dental Weekly*, offers the following to those who wish a quick method of flasking artificial dentures: After mixing enough plaster to fill the flask, fill one-half and put in the plaster cast on plate, shape the surrounding soft plaster so as to have no undercuts. Cover this filled half with a piece of tissue or bibulous paper, brush it over with soap-solution, put on the ring of the other half, fill up with the remaining plaster while yet soft, and put on the top of the flask. It can be opened as readily as if two mixes had been made.

---

Local Treatment for Pyorrhœa Alveolaris.—After thorough removal of deposits, Dr. Younger says he uses lactic acid to create an irritation to excite granulation, protecting surrounding tissues by applying glycerin and covering with cotton. He floods the pockets, leaving the lactic acid there. At a subsequent sitting chlorate of potash, as strong as can be borne, will be found very soothing. (He only uses tincture of iodine in cases of excessive inflammation.) Deposits should be *thoroughly* removed, and pockets properly cleansed of blood, serum, etc., when one application, "once for all," will be all that is required. Failure in these respects entails failure in treatment. Success is obtained in ninety-six per cent. of cases. Lining membrane will be exfoliated, contraction follow, and gum cling closely to the root again. If syringe-point can be introduced at end of week, treatment has not been thorough and must be repeated. Lactic acid is kept in small test-tube, and can be liquefied over alcohol flame. If not warm, it causes too much pain.—*Stomatologist.*

